# Regional variability in use of a novel assessment of thoracolumbar spine fractures: United States versus international surgeons

**DOI:** 10.1186/1749-7922-2-24

**Published:** 2007-09-07

**Authors:** John Ratliff, Neel Anand, Alexander R Vaccaro, Moe R Lim, Joon Y Lee, Paul Arnold, James S Harrop, Raja Rampersaud, Christopher M Bono, Ralf H Gahr, Trauma Study Group Spine

**Affiliations:** 1 Department of Orthopaedics, Thomas Jefferson University Hospital, Philadelphia, USA; 2 Department of Orthpaedics, Cedars Sinai Medical Center, Los Angeles, USA; 3 Department of Orthpaedics University of North Carolina, Raleigh, USA; 4 Department of Orthpaedics University of Pittsburgh, Pittsburgh, USA; 5 Department of Neurosurgery, Kansas University, Kansas City, USA; 6 Department of Orthopaedic Surgery, University of Toronto, Toronto, Ontario, Canada; 7 Dept. of Othopaedic Surgery, Brigham and Women's Hospital, Boston, USA; 8 Dept. of Othopaedic Surgery, Trauma Center St. Georg, Leipzig, Germany

## Abstract

**Background:**

Considerable variability exists in clinical approaches to thoracolumbar fractures. Controversy in evaluation and nomenclature contribute to this confusion, with significant differences found between physicians, between different specialties, and in different geographic regions. A new classification system for thoracolumbar injuries, the Thoracolumbar Injury Severity Score (TLISS), was recently described by Vaccaro. No assessment of regional differences has been described. We report regional variability in use of the TLISS system between United States and non-US surgeons.

**Methods:**

Twenty-eight spine surgeons (8 neurosurgeons and 20 orthopedic surgeons) reviewed 56 clinical thoracolumbar injury case histories, which included pertinent imaging studies. Cases were classified and scored using the TLISS system. After a three month period, the case histories were re-ordered and the physicians repeated the exercise; 22 physicians completed both surveys and were used to assess intra-rater reliability. The reliability and treatment validity of the TLISS was assessed. Surgeons were grouped into US (n = 15) and non-US (n = 13) cohorts. Inter-rater (both within and between different geographic groups) and intra-rater reliability was assessed by percent agreement, Cohen's kappa, kappa with linear weighting, and Spearman's rank-order correlation.

**Conclusion:**

Non-US surgeons were found to have greater inter-rater reliability in injury mechanism, while agreement on neurological status and posterior ligamentous complex integrity tended to be higher among US surgeons. Inter-rater agreement on management was moderate, although it tended to be higher in US-surgeons. Inter-rater agreement between US and non-US surgeons was similar to within group inter-rater agreement for all categories. While intra-rater agreement for mechanism tended to be higher among US surgeons, intra-rater reliability for neurological status and PLC was slightly higher among non-US surgeons. Intra-rater reliability for management was substantial in both US and non-US surgeons. The TLISS incorporates generally accepted features of spinal injury assessment into a simple patient evaluation tool. The management recommendation of the treatment algorithm component of the TLISS shows good inter-rater and substantial intra-rater reliability in both non-US and US based spine surgeons. The TLISS may improve communication between health providers and may contribute to more efficient management of thoracolumbar injuries.

## Background

Controversy persists with regard to treatment of thoracolumbar injuries. The diagnosis and definition of clinically significant spinal instability remains unclear and poses a source of frequent disagreement in the literature. Some authors note good clinical outcomes with non-operative treatment of these injuries; prospective studies demonstrate that many thoracolumbar fractures may be successfully treated non-operatively, with no benefit gained from adding surgical stabilization [1-3]. Some patients, however, ultimately fail conservative treatment, developing symptomatic late deformity or instability. Modern operative techniques allow for restoration of normal spinal alignment, correction of instability, and decompression of neural elements. Determining prospectively which patients are prone to developing instability and hence might benefit from surgical treatment remains contentious [4].

Adding to confusion over patient selection, no consensus exists as to choice of treatment in thoracolumbar injuries. The therapeutic approach to these patients is hampered by lack of accepted nomenclature and of a useful and clinically valid classification system for these injuries. While numerous classification systems have been devised, each poses problems in implementation [5]. Many systems are overly complex, limiting their utility. Others omit important portions of standard clinical decision making. Most classification schema fail to suggest treatment options [6].

**Figure 1:**
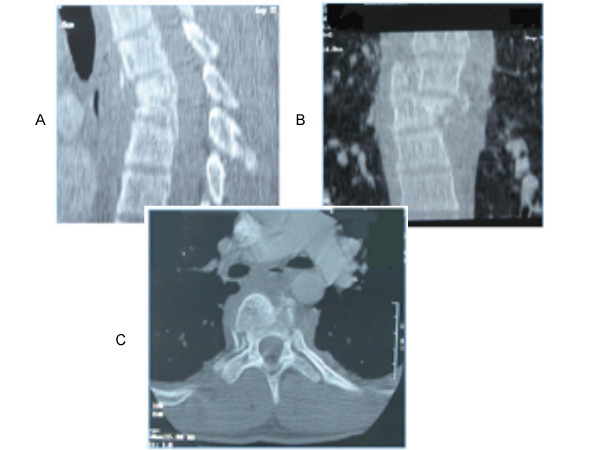
Illustrative case of TLISS use. Patient is an 18 y/o male who presents after a motor vehicle accident. Representative sagittal (A), coronal (B) and axial (C) computed tomography images were obtained. A compression fracture with angular deformity at T5 combined with a significant rotational injury is evident. Only the highest scoring injury, the translational/rotational score, is used for morphology (3 points). CT imaging suggests posterior ligamentous disruption due to severity of rotational deformity at the fracture site, and a palpable step between spinous processes on physical exam confirmed PLC injury (3 points). The patient was neurologically intact (0 points). The comprehensive score of 6 suggests operative therapy. An intact patient with disrupted PLC favors a posterior approach in the treatment algorithm [6]. The patient was treated with a multilevel posterior stabilization and fusion.

**Figure 2:**
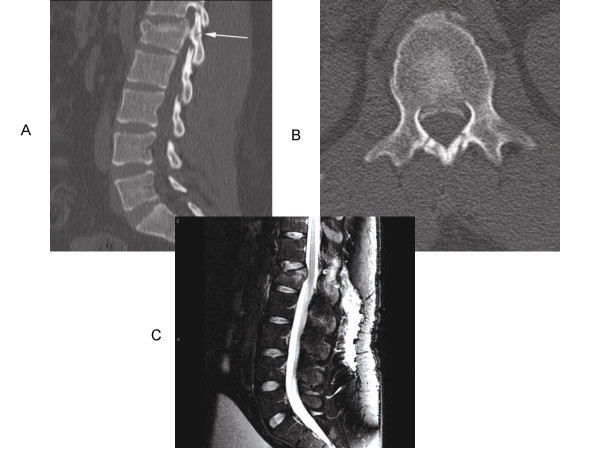
Second illustrative case of TLISS use. Patient is a 21 y/o male who presents after a motor vehicle accident. The patient was neurologically intact. Representative sagittal (A) and axial (B) computed tomography and sagittal T2-weighted magnetic resonance images (C) were obtained. A compression fracture with compromise of the superior endplate of L1 is found. Nondisplaced laminar fractures were present in the posterior elements bilaterally (Figure 2 A, white arrow). MRI imaging showed increased signal in the interspinous space, possibly indicating ligamentous injury and confirming involvement of posterior spinal elements. The patient receives one point for the compression fracture and an additional point for burst characteristics (posterior bony fractures). MRI suggests posterior ligamentous disruption (2 points). The patient was neurologically intact (0 points). The comprehensive score of 4 suggests either operative therapy or external orthosis may be used. We chose conservative treatment in this case.

A recently described treatment algorithm may aid in treatment of these patients. The Thoracolumbar Injury Severity Score (TLISS) assesses injuries based on three criteria: the mechanism of injury based upon radiographic assessment, patient neurological status, and the integrity of the posterior ligamentous complex (PLC) [4]. TLISS was developed by a group of 40 spine experts from 15 trauma centers in the United States, Canada, Australia, Germany, Mexico, France, Sweden, India, and the Netherlands. Relevant literature on thoracolumbar trauma, classification, and treatment was reviewed. A classification scheme and treatment algorithm were described (Figures 1 and 2) [7-9].

The TLISS algorithm has shown high initial treatment decision validity, with greater than 92% of surveyed surgeons agreeing with the algorithm's treatment recommendation (operative versus nonoperative) [10]. Initial testing showed poor reliability for the injury mechanism sub-score of the measure. Evaluation of early TLISS reliability assessments as well as surveys of the Spine Trauma Study Group led to modification of the protocol, with greater concentration upon injury morphology and de-emphasis of injury mechanism. A separate system, the Thoracolumbar Injury Severity and Classification Score (TLICS), has been recently forwarded [10]. Good validity in different specialties and different training levels have been described, with similarly adequate reliability [2,11]. Validation across geographic boundaries has not been assessed.

Considerable geographic variation is consistently reported in surgical treatment of spinal disorders. A universal rating scale and treatment algorithm for thoracolumbar injuries must overcome these regional variabilities and demonstrate acceptable reliability and validity regardless of locale. We compare the inter- and intra-rater reliability of the TLISS within and between US and non-US surgeons. We demonstrate moderate to substantial reliability in the use of the scale and high treatment validity, as assessed by surgeon agreement with the algorithm's management recommendation. The TLISS is a promising tool in the evaluation of spine trauma patients.

## Methods

Forty-eight trauma spine surgeons, including both neurosurgeons and orthopedic surgeons, participated in a comprehensive review and analysis of historical and contemporary thoracolumbar injury classification schemes, eventually leading to development of the TLISS clinical tool [8-10].

In order to validate the management recommendations of the TLISS algorithm, a booklet of 56 thoracolumbar traumatic injury case studies was prepared. The case vignettes detailed the patient's age, description of the traumatic injury, and neurological exam. Imaging studies, including plain radiographs, CT, and MR (sagittal T_2_-weighted images), were included. The cases were distributed to surgeons for classification and grading using the TLISS algorithm. The final severity score was used to determine the recommendation for nonoperative or operative treatment according to the treatment algorithm described in Table 1. Twenty eight surgeons completed the vignettes. The results were analyzed to determine inter-rater reliability, and percent agreement with the final treatment recommendations.

**Table 1:** Management and choice of approach

**Management**	**Points**	
Nonoperative	Less than 3	
Nonoperative or Operative	4	
Operative	Greater than 4	

**Operative Approach Algorithm**		

**Neurological Status**	**PLC Intact**	**PLC Disrupted**

Intact	Posterior approach	Posterior approach
Root Injury	Posterior approach	Posterior approach
Incomplete cord or cauda equina	Anterior approach	Combined
Complete cord or cauda equina	Posterior or combined	Posterior or combined

Summation of the TLISS points are referred to a management algorithm illustrated above. If the summation is less than 3 points than typically non-operative treatment is suggested, points greater then 5 suggest severe instability and the need for operative treatment. In addition a summation of four pints required further analysis on the patient concurrent modifiers and medical comorbidities. Taken from Vaccaro AR, Lehman RA, et al. and Harrop J, Vaccaro AR, et al [8,21].

Three months later, the numerical order of the cases was scrambled and pamphlets were redistributed. Twenty-two of the original 48 surgeons who participated in the development of the classification system completed both surveys. Their results were analyzed to determine intra-rater reliability. To assess possible regional differences in the validity and reliability of this system, physicians were grouped into US and non-US cohorts. The US cohort included surgeons from a variety of trauma centers. The international group included surgeons from Canada, Australia, Germany, Mexico, France, Sweden, India, and the Netherlands. The data were then analyzed using SPSS^® ^and Analyze IT^® ^software to determine percent agreement, unweighted Cohen's kappa, kappa with linear weighting, and Spearman's rank order correlation. The Cohen's kappa value was defined as the observer agreement (Pa) minus the chance agreement (Pc) divided by the maximum possible agreement that is not related to chance (1-Pc): kappa = (Pa – Pc)/(1-Pc). The kappa values (Table 2) obtained may range from – 1.0 (complete disagreement) through 0 (chance agreement) to 1.0 (perfect agreement) [23]. A guideline for interpreting Cohen's kappa values is summarized in Table 2. For significance tests, all unweighted coefficients were converted into Fisher's z-scores, and the difference in z-scores was divided by standard error. A level was set at 0.05 (ΔZ/SE ≥ 1.96).

**Table 2:** Interpretation of kappa statistics [23]

**Kappa**	**Agreement**
< 0	Less than chance agreement
0.01–0.20	Slight agreement
0.21–0.40	Fair agreement
0.41–0.60	Moderate agreement
0.61–0.80	Substantial agreement
0.81–0.99	Nearly perfect agreement

## Results

Inter- and intra-rater agreement between the cohorts is reviewed in Tables 3 and 4. General results of kappa scoring between the groups has been previously reviewed [10,12-14]. Non-US spine surgeons had greater inter-rater reliability on mechanism sub-score (p < 0.05 as assessed by % agreement), while US surgeons had greater inter-rater reliability on neuro status (p < 0.05 as assessed by % agreement and Spearman's r) and PLC integrity (p < 0.05 as assessed by Spearman's r). Intergroup (between USA and international) reliability was similar to within group inter-rater reliability in all parts of the TLISS scoring, indicating that the two groups agreed about as often as individual members within each cohort agreed amongst themselves.

**Table 3:** US versus non-US inter-rater agreement

	***Percent Agreement***	***Cohen's Kappa***	***Weighted Kappa***	***Spearman's Rank Order***
	**Mechanism**			

USA (Within Group)	47.2	0.262	0.276	0.376
Non-USA (Within Group)	55.7*	0.351	0.300	0.362
Between USA and Non-USA	52.1	0.313	0.297	0.38

	**Neurological Status**			

USA (Within Group)	97.9*	0.963	0.976	0.988*
Non-USA (Within Group)	94.9	0.911	0.958	0.981
Between USA and Non-USA	96.3	0.936	0.967	0.984

	**PLC Integrity**			

USA (Within Group)	62.1	0.373	0.464	0.551*
Non-USA (Within Group)	60.6	0.336	0.425	0.503
Between USA and Non-USA	61.8	0.361	0.45	0.532

	**Total TLISS**			

USA (Within Group)	31.8	0.23	0.532	0.719*
Non-USA (Within Group)	36.9*	0.28	0.528	0.697
Between USA and Non-USA	35.5	0.267	0.538	0.713

	**Management**			

USA (Within Group)	75.7*	0.561	0.516	0.541*
Non-USA (Within Group)	72.3	0.506	0.462	0.487
**Between USA and Non-USA**	74.2	0.536	0.498	0.527

Inter-Rater agreement within and between USA and non-USA surgeons.

*p < 0.05 for difference between USA and non-USA surgeons. For significance tests, all unweighted coefficients were converted into Fisher's z-scores, and the difference in z-scores was divided by standard error.

A level was set at 0.05 (ΔZ/SE ≥ 1.96).

**Table 4:** US versus non-US intra-rater agreement

	***Percent Agreement***	***Cohen's Kappa***	***Weighted Kappa***	***Spearman's Rank Order***
	**Mechanism**			

USA	62.2*	0.454	0.465	0.561
Non-USA	56.0	0.398	0.519	0.605

	**Neurological Status**			

USA	87.6	0.781	0.803	0.825
Non-USA	96.2*	0.933	0.956	0.982*

	**PLC Integrity**			

USA	67.4	0.455	0.519	0.578
Non-USA	69.7	0.507	0.610	0.685*

	**Total TLISS**			

USA	43.3	0.354	0.588	0.726
Non-USA	41.4	0.342	0.658	0.825*

	**Management**			

USA	78.8	0.617	0.582	0.604
**Non-USA**	77.6	0.610	0.577	0.609

Intra-Rater agreement within USA and non-USA surgeons.

*p < 0.05 for difference between USA and non-USA surgeons. For significance tests, all unweighted coefficients were converted into Fisher's z-scores, and the difference in z-scores was divided by standard error.

A level was set at 0.05 (ΔZ/SE ≥ 1.96).

Absolute inter-rater agreement among non-US surgeons on the final TLISS score was greater (p < 0.05), but total TLISS scores better correlated among US surgeons (p < 0.05) (Table 3). With regard to the algorithm's final recommendation for treatment (operative vs. non-operative), inter-rater agreement within the US physician group and non-US group was 75.7% (Cohen's kappa .561) and 72.3% (Cohen's kappa .506), respectively (Table 3). Inter-rater agreement on management between the groups was 74.2% (Cohen's kappa .536). Between the two groups, greatest agreement was found in assessment of neurological status (96.3% agreement, Cohen's kappa .936) (Table 3).

Intra-rater agreement was higher among US surgeons on mechanism, whereas intra-rater agreement was higher among non-US surgeons for neurological status, PLC integrity, and total TLISS score. These differences reached statistical significance as assessed by % agreement for mechanism and neurological status. Differences in intra-rater correlation reached statistical significance on neurological status, PLC, and total TLISS (Table 4). Intra-rater reliability on management in the two cohorts was similar, with 78.8% intra-rater agreement in US surgeons (Cohen's kappa .62) and 77.6% in non-US surgeons (Cohen's kappa .61). US surgeons agreed with the management recommendation of the TLISS in 93.4% of the cases and non-US surgeons agreed with the algorithm in 91.3% of the cases.

## Discussion

### Thoracolumbar classification schema

Initial attempts at thoracolumbar fracture classification were made by Bohler in 1930, who classified fractures into five injury types based on anatomic appearance and mechanism [12]. The modern era of fracture classification benefited greatly from availability of CT scanning; advances in imaging led Denis to develop a three-column model of spinal stability, modifying the two-column approach of Holdsworth and Louis [7,15]. Magerl et al. forwarded the AO classification, using a mechanistic approach to divide fractures into a total of 53 potential patterns based upon 3 injury categories and 3 tiers of subcatagorization [16]. A separate load-sharing classification of spinal injury has also been described [17].

The most commonly used systems are Denis' three-column model of spinal stability and the AO classification. Both have significant problems. The AO system has poor inter- and intra-observer agreement [6,9]. Use of 53 different fracture patterns is unwieldy and appears counterintuitive. This makes routine clinical use of the scale impractical. The Denis system may oversimplify complex fractures, and may not accurately assess need for operative intervention [12].

### TLISS clinical algorithm

The TLISS clinical algorithm assesses thoracolumbar injuries based upon three accepted clinical decision making criteria: 1. Mechanism of injury as determined by imaging studies, 2. Integrity of the PLC, and 3. Patient neurological status. These criteria were thought to be independent predictors of patient clinical outcome. Subgroups for scoring were developed within each component. Points are assigned in the treatment algorithm cumulatively for each criterion. Final recommendation for treatment is based upon final injury score (1 and 2).

#### Mechanism of injury

The mechanism of injury describes fracture pattern based upon three general descriptions, similar to the AO thoracolumbar injury classification: 1. compression, 2. translation/rotation, and 3. distraction (Table 5). Angulation at the fracture site for compression injuries indicates greater instability, and separately may add 1 point to final TLICS score. Complex fractures may combine more than one of the three basic morphologic elements. In these cases, only the highest category is scored.

**Table 5:** Thoracolumbar injury severity score (TLISS)

**Mechanism**	**Qualifier**	**Points**
Compression	None	1
Compression	Lateral Angulation >15°	1
Compression	Burst	1
Translational/Rotational		3
Distraction		4

**Neurological Exam**	**Qualifier**	**Points**

Intact		0
Nerve Root		2
Cord, conus medullaris	Incomplete	3
Cord, conus medullaris	Complete	2
Cauda Equina		3

**Integrity of the Posterior Ligamentous Complex**		**Points**

Intact		0
Injury Suspected/Indeterminate		2
Injured		3

Thoracolumbar injury severity score (TLISS) illustrating three major categories of mechanism of injury, neurological involvement and posterior ligamentous complex with associated grading points. Taken from Harrop J, Vaccaro AR, et al [8].

#### Integrity of the PLC

The PLC is composed of the ligamentum flavum, the facet joint capsules, and the interspinous and superspinous ligaments. The PLC is quantified in the TLICS as intact, indeterminate, or disrupted (Table 5). Imaging via MRI, CT, plain films, and physical exam (detecting a palpable gap between spinous processes) are used to evaluate the PLC.

#### Neurological status

Presence or absence of neurological deficit is an independent indicator of the severity of thoracolumbar injury. More severe injuries merit higher scores, with incomplete spinal cord and cauda equina injuries scoring highest in the algorithm (Table 5).

The injury score is obtained via summation of individual elements. A cumulative score of 3 or less suggests a non-operative injury, while a score of 5 or greater suggests surgical intervention may be necessary (Table 1). Scores of 4 are indeterminate, and may be treated surgically or conservatively [4,6,12]. Illustrative cases are reviewed in Figures 1 and 2.

### Reliability and validity of the TLISS

The TLISS scale has been evaluated for both inter- and intra-rater reliability. Acceptable reliability was found and surgeons agreed with the algorithm's treatment recommendation in greater than 90% of cases [10]. These findings indicate the scale produces internally reliable ratings of injury severity and treatment recommendations that are valid with respect to the rating surgeons' clinical approaches [10]. Substantial reliability has been previously demonstrated within a variety of specialties and training levels, including spine fellows, attending spine surgeons, neurologists, and physiatry physicians [13]. Greater than 90% of surgeons in each specialty were found to agree with the TLISS management recommendations [14], and the same trend of outstanding construct validity is reported here when comparing US and non-US surgeons.

In contradistinction to other classification schemes, the TLISS has demonstrated acceptable intra- and inter-rater reliability and appears usable across specialty boundaries. This manuscript is the first to assess geographic differences in approach to thoracolumbar injuries using the TLISS assessment tool.

### Geography and spine surgery

As noted by Seidenwurm, "medicine is evidently a local phenomenon" [18]. Geographic influences on choice of surgical and medical therapies are significant. Geographic location consistently predicts yearly rates of spine surgery [19]. Authors have noted that rates of back surgery in the United States are 40% higher than other countries, and five-fold higher than comparable rates in England and Scotland. Rates of surgery are noted to increase linearly with supply of orthopedic and neurosurgical spine surgeons [8].

Other authors have noted significant regional differences in availability and utilization of medical imaging, and correlated these findings with rates of elective spinal surgery [18,20]. Similar geographic variation occurs in coronary artery bypass graft procedures, general orthopedic procedures, and medical treatment of acute myocardial infarction [11]. Parallel findings in systems without financial incentive for clinical productivity would seem to indicate an intrinsic regional variability in health care use [21,22]. Geographic variation also is found in development and adoption of new technology [23].

Geographic differences persist in evaluation and management of traumatic injuries. In a multi-center review of traumatic spine injuries, no consensus was found as to optimal surgical timing [24]. Treatment approach, including imaging, seemed to vary by research site [24]. For the TLISS to be a useful paradigm for assessment and management of thoracolumbar injury, it must bridge these significant geographic differences in approach to spinal pathology. The system must demonstrate adequate international reliability.

### Domestic versus international reliability and validity of the TLISS

We compared reliability and validity of the TLISS in US and non-US surgeons, assessing both inter- and intra-rater reliability. Results are reviewed in Tables 3 and 4. Inter-rater agreement on management within the US physician group and non-US group was 75.7% and 72.3, respectively. Overall correlation on management between the groups was 74.2%, indicating adequate validity of the measure. The TLISS provides reliable and valid initial treatment recommendations, irrespective of rater geography.

These and similar results have lead to a recent modification of the TLISS system [6]. Inferring the mechanism of injury from initial imaging modalities may be difficult. In fact, this is the least reliable sub-score among both US and non-US surgeons. Hence, injury morphology has been substituted for injury mechanism. Injury morphology is based simply upon the appearance of the fracture or dislocation on imaging studies (plain film, CT, or MRI). The STSG has also endeavored to increase the reliability of the PLC sub-score. To this end, a series of studies have been undertaken to clearly define the principle indicators of PLC disruption on MRI. These definitions will be included in a revised classification system. This revised classification system is termed the Thoracolumbar Injury Classification and Severity Score (TLICS). Only the most severe (highest total points) injury morphology category is included in the scoring. Compression morphology garners 1 point, and an additional point is assigned for burst morphology. Three points are assigned for a translational/rotational morphology and 4 points for a distraction morphology. The descriptive "distraction" is only applied if there is objective imaging evidence of distraction present. Scores of the morphology subgroups are not additive if multiple morphologies are present. Studies are underway to further develop and validate this classification system.

## Conclusion

Controversy persists in management of thoracolumbar injuries. The TLISS clinical algorithm offers assessment of injury stability and aids in making treatment decisions. The scale has shown adequate reliability between and within different specialties. We show reliability and validity of the TLISS scale across geographic boundaries, comparing US and non-US surgeons. Differences between these broad geographic groups were subtle, with inter-rater reliability between groups similar to reliability within groups. This suggests that the TLISS may help unifying clinical decision making in thoracolumbar trauma.

## Competing interests

The author(s) declare that they have no competing interests.

## Authors' contributions

JR assembled cases for review and led the manuscript preparation effort the manuscript. NA participated in writing the manuscript and interpreting the results of the reliability data. AR conceived of and designed the study, and led the development of the TLISS. ML assisted in data analysis and manuscript preparation, including writing of the methods section. JL led data collection and analysis and assisted in manuscript preparation. PA performed the literature review and assisted in manuscript preparation. JH was a leader in the development of the TLISS and wrote the discussion section of the paper. RR was a leader in the development of the TLISS and assisted in manuscript preparation. CB was a leader in the development of the TLISS and assisted in manuscript preparation. RG was a leader in the development of the TLISS and drove European participation in this project. The entire STSG participated in the evolution of this classification system and served as case raters for this reliability study. All authors reviewed and approved of the final manuscript.
